# Clean Energy Based Multigeneration System for Sustainable Cities: Thermodynamic, and Stability Analyses

**DOI:** 10.3390/membranes13030358

**Published:** 2023-03-20

**Authors:** Uzair Bhatti, Hamza Aamir, Khurram Kamal, Tahir Abdul Hussain Ratlamwala, Fahad Alqahtani, Mohammed Alkahtani, Emad Mohammad, Moath Alatefi

**Affiliations:** 1 Department of Engineering Sciences, National University of Sciences and Technology, Islamabad 44000, Pakistan; buzair6@gmail.com (U.B.); hamzaamir93772@gmail.com (H.A.); khurram.kamal@pnec.nust.edu.pk (K.K.); tahir.ratlamwala@pnec.nust.edu.pk (T.A.H.R.); 2 Industrial Engineering Department, College of Engineering, King Saud University, Riyadh 11421, Saudi Arabia; moalkahtani@ksu.edu.sa (M.A.); 441106540@student.ksu.edu.sa (M.A.); 3 Electrical Engineering Department, College of Engineering, King Saud University, Riyadh 11421, Saudi Arabia; 442106835@student.ksu.edu.sa

**Keywords:** multigeneration, renewable energy, hydrogen, Simulink, root locus, stability analysis

## Abstract

This paper concerns the development and analysis of multigeneration systems based on hybrid sources such as biomass and wind. Industry requires different types of sources to provide several outputs, so the goal of this research was to fulfill the industrial requirement with optimization. The multigeneration cycle supplies enough power to satiate energy demands, i.e., power, cooling, hydrogen, air conditioning, freshwater, hot water, and heating. For this, the multigeneration cycle was modeled in the Engineering Equation Solver (EES) and Simulink to obtain optimized results for the industry. Energy and exergy for the multigeneration cycle were determined to assess the performance of the cycle and to investigate the optimized results for the overall system. This study shows that for configuration selection and design, different thermodynamic, economic, and environmental aspects should be considered. Based on the results, the selection of the best location for this multigeneration system was made. Power output from the wind turbine was around 7 MW and from biogas 0.6 MW. The overall exergy efficiency of the multigeneration system was found to be 0.1401.

## 1. Introduction

Energy is an important part of national development and with the advancement of time, it is becoming a more important requirement. People are now looking for many new ways to increase energy production, and with fossil fuels decreasing day by day, renewable energy is becoming a better option going forward. Being the only means of clean, green, and infinite energy, renewable energy mainly has solar, wind, biomass, geothermal, and hydro as the main sources [1]. A multigeneration energy system is used to utilize more than three sources. A multigeneration energy system provides higher efficiency than the combined equal and separate units. When a multigeneration system uses renewable energy, it combines clean and green energy with higher efficiency. In addition, it also supplies different needs of the public unit such as the neighborhood. Using an energy-based multigeneration system, it has been analyzed that exergy efficiency varies from 55% to 65% depending on the amount of cogeneration used. Murat Ozturk et al. [2] showed that, through the integration of various systems, multigeneration increases energy and exergy efficiencies. Furthermore, it has been determined that, due to renewable energy-based multigeneration, the fuel price decreases, as well as harmful pollutant emissions, compared to conventional systems [3].

The objectives of this paper include first proposing and assessing a new integrated multigeneration system using biomass and wind energy with energy and exergy analyses, including the determination of overall energy and exergy efficiencies of the multigeneration system and its subsystems; and second carrying out a parametric study to determine the effects of various parameters on the overall energy and exergy efficiencies of the multigeneration system and its subsystems. Challenges faced in this research and study include the difficulty of scheduling and modeling the sources; wind, and biomass in this case. Wind energy is not constant throughout the year. It changes with the temperature and season. However, we can use the average wind speed for analysis. In addition, for biomass, the waste disposal can vary from day to day, but through observation, average waste disposal can be found to be used as a reference for calculation.

Renewable energy can be obtained from the biological remains of the living. It is known as biomass, and it can be a great way to get the most out of waste. All living things present on Earth are comprised of biomass. It can either be converted into other forms such as biofuels or it can be used directly [4]. Biomass is used currently for heating, electricity, and cooling [5]. To obtain large-scale utilization, biomass is directly burned using the combustion process of coal, which is considered the most common method of conversion [6]. There are other methods more efficiently feasible than the combustion process for power generation, which include thermochemical conversion technologies such as gasification and pyrolysis. Economically these technologies are not viable for large-scale utilization, or they lack maturity and reliability [7]. 

A 2-kW biomass-fired micro-scale CHP system was studied by Liu et al. [8] where they used the organic Rankine cycle with three organic working fluids. Efficiency variation was also assessed by them through a parametric study of the CHP system with selected design parameters. Using biomass as a supplementary fuel, Gnanapragasam et al. [9] studied the optimum conditions for a natural gas combined cycle power generation system. In addition, biomass was also used to increase the temperature of flue gases in a supplementary firing unit. Al-Sulaiman et al. [10] reported energy and exergy analyses of a biomass trigeneration system using an organic Rankine cycle. They also performed a comprehensive parametric study of the system and concluded that there is a significant improvement when trigeneration is used in place of only electrical generation.

A biomass-based integrated comprehensive system with hydrogen production was proposed by Safari and Dincer [11] and production rates for power hydrogen (0.347 kg/h), freshwater (0.94 kg/s), and hot water (1.82 kg/s) were 1102 K W/h. Ahmadi et al. [1], developed an integrated bio-mass multi-generation system and concluded that when the system is multi-generating, the potential for CO_2_ emission reduction is extensive. Khalid et al. [12], integrated solar and biomass in the multi-generation system, Moradi et al. [13], considered bio-mass gasification only, and Paakkonen and Joronen [14], restudied the feasibility of a biomass-integrated combined heat and power system. Studies in [12,13,14], revealed that biomass and biogas are highly feasible for comprehensive and multi-generation systems with maximum energy and exergy efficiency of 72.5% and 30.44% and can be derived from environmental wastes such as chicken manure [15,16], maize silage [17], rice husk [18], etc. Sevinchan et al. [19], developed a multi-generation system powered by maize silage and chicken manure. Rice husk was hybridized with solar energy to power another multi-generation system developed in the literature [20]. The ammonia and hydrogen productions by their system [20] were 79 g/s and 20 g/s. Solar and wind are also considered the most commonly used RE sources of power, co-generation, and multi-generation. Ozlu and Dincer [21], analyzed a multi-generation system based on solar and wind energy and reported the overall energy and exergy efficiencies of the systems to be 43% and 65%. 

The wind is one of the most abundant renewable energy sources used nowadays. The wind energy can be extracted by a wind energy conversion system (WECS), which is composed of a wind turbine, electric generator, power electronic converter, and the corresponding control system. Wind energy at different wind velocities has to be converted to electric power at the given grid frequency. To choose other components of the wind energy conversion system (WECS), the speed control strategy should be known. Wind turbine configurations are based on different categories, i.e., horizontal and vertical axes, number of blades, and power rating. This paper used a wind turbine of a horizontal-axis type, having three blades and a power output of 0.56 MW [21]. Extended energy, exergy, and economic analyses of a wind turbine were presented in another study and the maximum exergy efficiency was 10.8%. The exergy analysis of a CHP system integrated with wind turbines was presented by Mohammadi et al. [22], and the operating parameters of a hybrid wind–hydrogen system were analyzed energetically and exegetically by Fakehi et al. [23]. 

Integration of a wind generation system and electrical system development are associated with both benefits and costs. Compromise has been made either in cost or benefit. For example, wind generation required more investment than that of conventional gas or coal plants. Whereas the energy generation from wind saves fuel consumption hence reducing the cost of system operation.

There are some issues related to the integration of wind power into a system. Although the energy produced by a large conventional plant may be displaced by that of wind generation, concerns over system operation costs are focused on whether wind generation will be able to replace the capacity and flexibility of conventional generation plants. Moreover, another important factor is the location of these new sources in assessing the impacts on the transmission and distribution network infrastructure. Wind turbines generally cannot provide a range of system support services (e.g., voltage and frequency regulation) that are provided by thermal and hydro plants.

Wind cannot be considered the sole power generator. It is necessary to retain a portion of conventional plants for backup or to ensure security, especially under conditions of high demand and low wind. This concludes that wind power is variable and not easy to predict, various forms of additional reserves will need to be introduced to maintain a constant balance between supply and demand. So, a conventional generation system cannot be displaced by a wind generation system. 

All these studies conclude that a wind energy integrated system is feasible and enhances the performance of the system. The main challenge in this comprehensive system is that most research has been presented on the steady-state condition which gives no insight into the performance of the system while the thermodynamic analysis of wind and biomass for a comprehensive energy system has also been limited. So, the gap can be bridged by developing a novelty for this system. The system will be analyzed in steady-state and time-based conditions and assessing the system by using the Engineering Equation Solver (EES) computer program and Simulink.

## 2. Materials and Methods

### 2.1. Developing a Schematic Diagram 

For this study, a multigeneration cycle had to be designed to provide power, cooling, heating, freshwater, hydrogen gas, desalinated water, and conditioned air as an output. Multigeneration had to be powered using biogas generated from cow manure as well as wind turbine power. The main aim of this study was to develop a model of the multigeneration cycle using the EES and Simulink for thermodynamic analysis. Finally, the model developed had to be tuned to obtain a steady state for the cycles of the multigeneration system.

**Figure 1:**
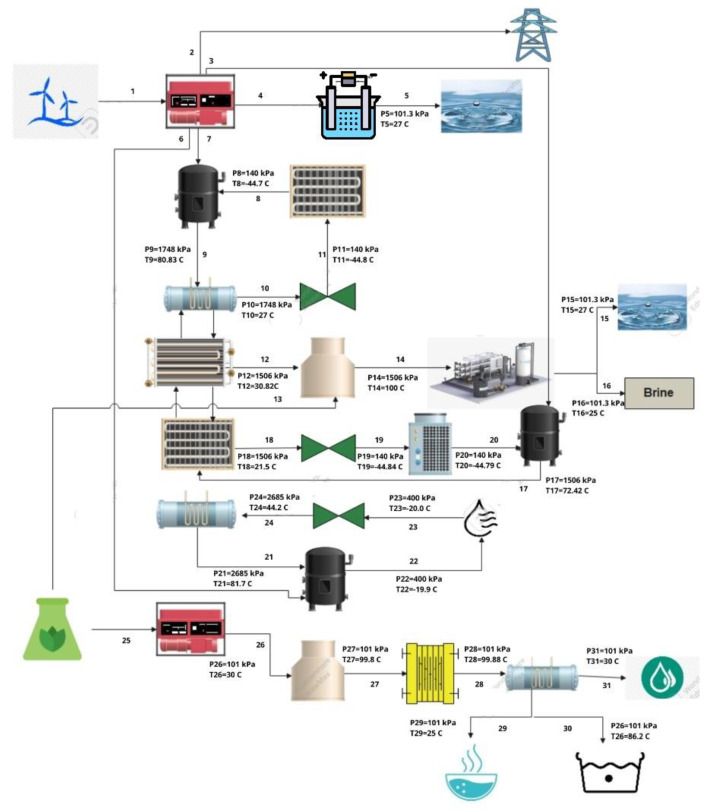
Schematic diagram.

Figure 1 shows the system which utilizes wind as a primary source and biomass as a secondary source. The system produces power, cooling, heating, fresh water, hydrogen, hot water, and conditioned air as its output. The components used in this system include generator, compressor, condenser, pump, turbine, expansion valve, electrolyser, evaporator, boiler, heat exchanger, desalination plant, water treatment plant, heat pump, air-conditioning, and refrigeration cycles. Schematic is labelled with total of 31 different states points for which the working fluid or substance for which pressure, temperature, internal energy, enthalpy and entropy had to be determined. All these components work in a flow system to produce the desired output. The schematic of this system is illustrated in Figure 1. 

The atmospheric conditions are 28 °C and 101 KPa. Wind velocity is 8 m/s having a power of 1.293 × 10^6^ used to drive the wind turbine that ultimately drives the generator. The wind turbine gives a power output of 1.033 × 10^7^ and this power output is used to produce electricity only. The electrolysis process is performed by using heat from the generator to produce H_2_ and O_2_. The remaining heat of the generator is used to drive the vapor compression cycle. The working fluid used in the vapor compression cycle is R410, the compressor operates at 80.83 °C, the condenser operates at 27 °C, expansion valve, and the evaporator operates at −44.84 °C. The vapor compression cycle gives cooling as an output. The heat released by the condenser is absorbed by the heat exchanger. The heat exchanger provides water with the heat to initially increase its temperature to 30 °C. The heat exchangers used for this multigeneration system are parallel flow heat exchangers. This heat is utilized by the boiler. The boiler obtains two sources of heat, one from the heat exchanger and the other from biomass. The desalination plant utilizes the steam of the boiler to give fresh water and brine. Thermal desalination is performed using the boiler. Both the boiler and condenser operate at an atmospheric pressure of 101.325 KPa. Water enters the boiler at around 30 °C and leaves as steam. It is then condensed to around room temperature. The temperatures of fresh water and brine are 27 °C and 25 °C. 

The heat released by the condenser of the air conditioning cycle is absorbed by the evaporator. The operating temperature of the evaporator is 21.5 °C. The evaporator is also connected to the compressor. Then, the expansion valve opens and the heat pump produces heat. The heat pump then provides the output heat that is used for heating. The compressor of the air-conditioning cycle obtains heat from the wind turbine generator. The air conditioning cycle provides the conditioned air that is used for space heating.

Biomass is used as a fuel for the generator to produce power. The operating condition of the generator is 30 °C. This power is used by the boiler to produce steam. The system heat exchanger absorbs heat from the boiler and gives that heat to the condenser. The condenser obtains cold water from one of its inlets and water leaves as hot water from one of the outlets. After the condensation of steam, treated water is obtained from the system. Treated and hot water are obtained at temperatures of 30 °C and 25 °C respectively. Above all the processes work in rhythm to produce the desired output.

For this multigeneration cycle, several parameters were required. Most of the parameters required were operating intensive properties for the cycle or constraints for the power generation such as wind and biogas. Table 1 shows the parameters required to analyze the whole multigeneration cycle. 

### 2.2. Developing Input Source/Components/Process for the Multigeneration Cycle 

**Figure 2:**
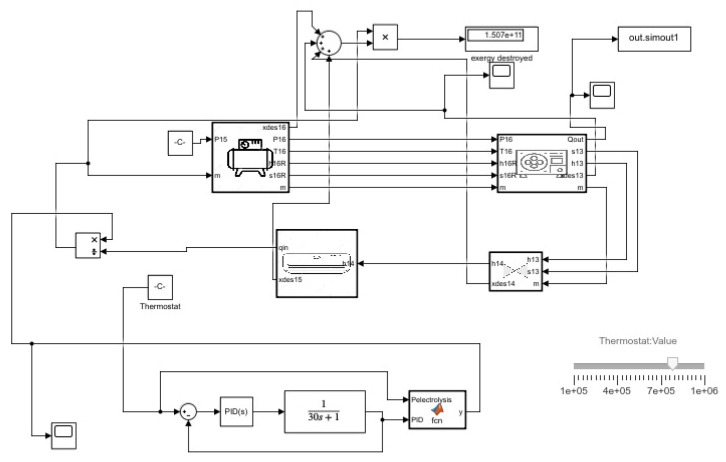
Airconditioning cycle Simulink model.

In the air condition cycle, as shown in Figure 2, analysis was carried out based on the application of the first-order transfer function on different mass values with pressure ranging from 0.2 MPa to 1 MPa. Using the thermostat, the mass flow was controlled by integrating the thermostat between the condenser outlet and the evaporator inlet. The mass flow rate was calculated by using Qin and thus it could be determined how much mass flow rate could be allowed for the conditioning of air.

**Figure 3:**
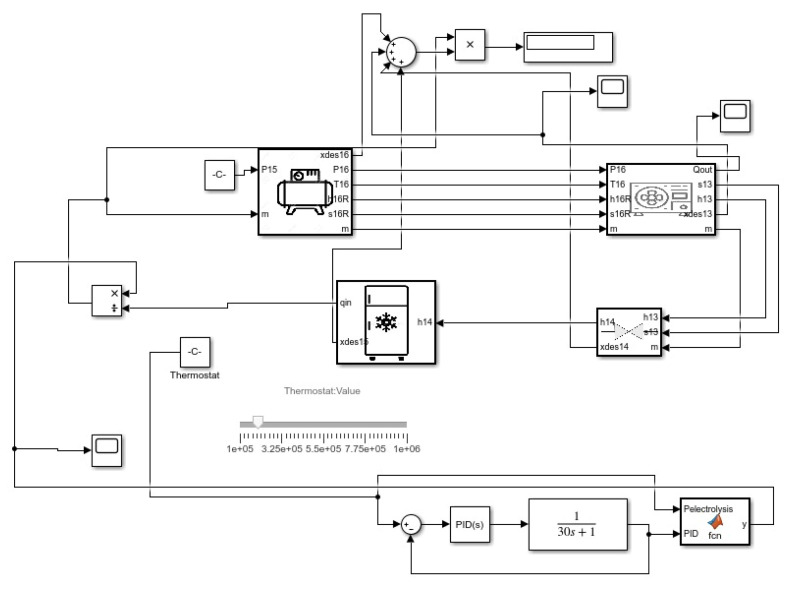
Refrigeration cycle Simulink model.

In the refrigeration process, as shown in Figure 3, the same was conducted based on the application of the first-order transfer function on different mass values with pressure ranging from 0.14 MPa to 0.8 MPa. Using the thermostat, the mass flow was controlled by integrating the thermostat between the condenser outlet and the evaporator inlet. The same method was applied to find the mass flow rate which is by calculation of the Qin of the evaporator and then controlling the mass flow of the refrigerant to obtain the desired cooling.

**Figure 4:**
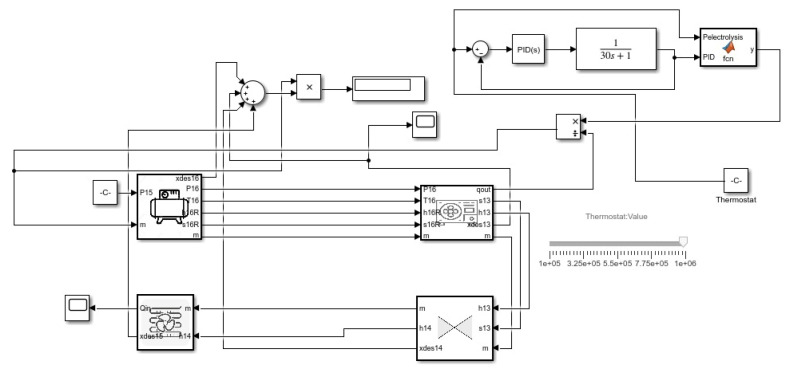
Heat pump Simulink model.

Almost the same method was applied to the heat pump as shown in Figure 4. Coolprop library was used, and the first order transfer function performed on different mass values: pressure ranges from 0.14 MPa and 0.8 Mpa in the heat pumping also. A thermostat was integrated between the condenser and evaporator, but the only difference observed here is that the useful heat is at the condenser. This heat is used to determine the mass and then it can be varied by changing the mass flow.

**Figure 5:**
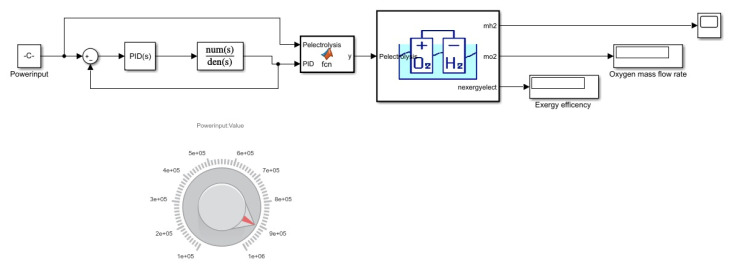
Electrolysis Simulink model.

Electrolysis requires the second-order transfer function because it depends on two variables which are the battery response and the hydrogen production rate as shown in Figure 5. When the power is supplied, electrolysis provides the current based on the given power. This current then determines the amount of oxygen and hydrogen produced. So, the production can be varied depending on the two variables that are changed to obtain the best output.

**Figure 6:**
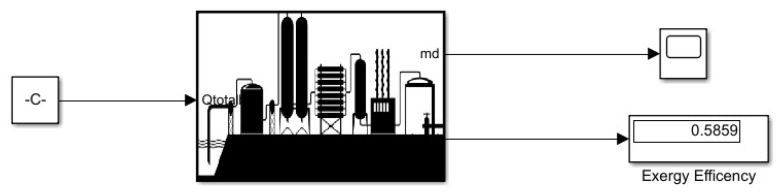
Desalination Simulink model.

As in Figure 6 the desalination system is based on the third-order transfer function because it has three different processes which are boiling, condensation, and storage. Output is the treated water against the given power; the water is first boiled which releases brine and steam and then the water vapor is condensed and stored in the collector.

**Figure 7:**
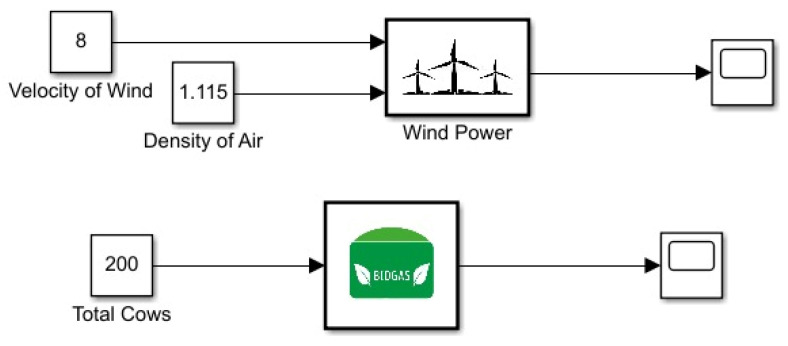
Power generation Simulink model.

As in Figure 7 for the multigeneration system, two renewable energy systems were used, namely wind and biogas. The Betz law and kinetic energy conversion to electrical energy were used to determine total power generation for wind. Biogas energy production was modeled using the estimated power which could be generated from cow manure in a single day. Since the system does not contain major variables which vary with respect to time, for multigeneration sources modeling a zero-order system was assumed.

**Figure 8:**
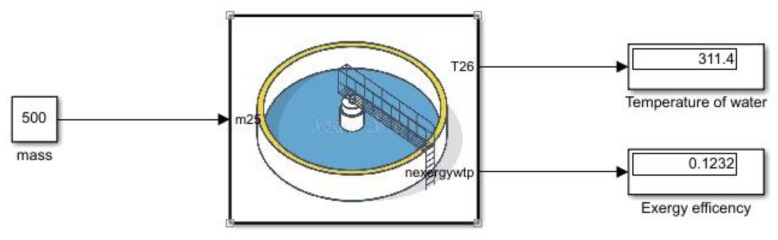
Water treatment Simulink model.

As in Figure 8 the hot water system is modeled with respect to time. The mass flow rate cannot be assumed to be zero if the mass flow rate is assumed to approximate zero or the slightest change of heat will result in temperature gain which is greater than zero which results in negative exergy destruction. Exergy destruction can never be negative so modeling steady-state response will result in unreasonable results.

### 2.3. Approach to Analysis 

Exergy can be found by determining the entropy generated between each component of the gas turbine. Exergy destroyed *X_des_* is found by multiplying the entropy generated with the outside temperature and exergy destruction can be determined. Where *Q_H_* is heat gained, *Q_L_* is heat released, *T_H_* and *T_L_* are the temperatures at the hot and cold source respectively from initial *i* to final state *f* as in Equations (1)–(3)

(1) $${\dot{X}}_{dest,\;i-f}=T_{0}{\dot{S}}_{gen,\;i-f}=T_{0}[\dot{m}\left(s_{f}-s_{i}\right)+\frac{{\dot{Q}}_{H}}{T_{H}}]$$

(2) $${\dot{X}}_{dest,\;i-f}=T_{0}{\dot{S}}_{gen,\;i-f}=T_{0}[\dot{m}\left(s_{f}-s_{i}\right)+\frac{{\dot{Q}}_{L}}{T_{L}}]$$

(3) $${\dot{X}}_{dest,\;i-f}=T_{0}{\dot{S}}_{gen,\;i-f}=\dot{m}T_{0}\left(s_{f}-s_{i}\right)$$

Exergetic efficiency can be found using Equation (4)

(4) $$\eta_{II,des}=\frac{{\dot{X}}_{{\dot{Q}}_{L}}}{{\dot{W}}_{in}}=\frac{{\dot{W}}_{min,\;in}}{{\dot{W}}_{in}}=1-\frac{{\dot{X}}_{dest,total}}{{\dot{W}}_{in}}$$

Pelec showed the power transferred to carry out electrolysis as shown in Equation (5) where the current is produced. For the electrolysis, hydrogen and oxygen were produced by consuming the power generated by the generator. Where the amount of the current produced, *fo* is faraday’s constant. The equations used for the mass flow rate production of the hydrogen *mh*_2_ and oxygen *mo*_2_ gas are as follows:

(5) $$P_{elec}=I_{cur}\cdot \;V_{i}$$

(6) $$mh_{2}=\frac{JA_{i}}{2F}\;\cdot 0.002$$

(7) $$mo_{2}=\frac{JA_{i}}{4F}\;\cdot \;0.032$$

*P_wind_* is the maximum expected power generated from the wind turbine in Equation (8) while *u* is the collective efficiency of the wind turbine. Multiplying *P_wind_* with the collective efficiency and Bentz’s efficiency *P_output_* is determined in Equation (10) which is the actual power consumed.

(8) $$P_{wind}=0.5\rho v^{3}A$$

(9) $$u=\left(1-km\right)\left(1-ke\right)\left(1-kt\right)\left(1-kt\right)C_{p}$$

(10) $$P_{output}=n_{turb}\cdot u\cdot P_{wind}$$

Applying the approach for energy calculation for small-scale biogas production Equations (11)–(16) are used to determine the estimated amount of energy that could be extracted from the cow’s manure [25].

(11) $$T_{waste\;disposal}=totalcows\cdot \;Wastedisposal$$

(12) $$Total_{volatile\;solid}=Volatile_{solid\;cow}\cdot \;totalcows$$

(13) $$Conc_{vol\;solid}=\frac{Total_{volatile\;solid}}{Digestor_{vol}}$$

(14) $$S=\frac{Total_{volatile\;solid}}{Volume}$$

(15) $$G=Yieldfactor\cdot S\cdot \frac{Digestor_{vol}}{1000}$$

(16) $$Energy_{prod,days}=22.08\cdot G$$

According to the first law of thermodynamics energy balance, the equation has to be applied as in Equation (17). Ein represents the energy input while *E_out_* represents the energy output:

(17) $$E_{in}=E_{out}$$

Equations (18) and (19) represent the coefficient of performance for the refrigeration cycle and heat pump cycle:

(18) $$COP_{ref}=\frac{Q_{L}}{Q_{H}-Q_{L}}$$

(19) $$COP_{hp}=\;\frac{Q_{H}}{Q_{L}-Q_{H}}$$

## 3. Results

After conducting a literature review, and assuming parameters for the energy sources to power the multigeneration cycle; the multigeneration cycle was designed. It was powered by two sources wind and biogas. This cycle can produce hydrogen, provides desalinated water, treated water, and hot water for residential areas, while operating the refrigerators, air conditioner, and heat pump. The vapor compression cycle is used to operate the refrigeration cycle, air conditioning, and heat pump cycle. Single stage desalination is performed using a boiler operated by biogas energy, another boiler that receives heat input from the biogas is used to treat water and supply hot water to residential areas. Electrolysis produces hydrogen and oxygen gas which can be used for treating metals, processing food, refinement of petroleum, and as fuel.

**Figure 9:**
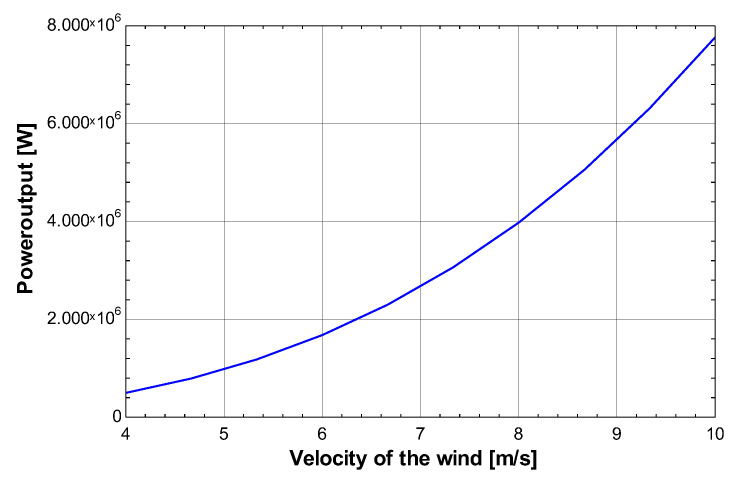
Effect of the velocity of air on wind power.

Figure 9 shows the relationship between the velocity of air and wind power in the wind cycle using the blue line. With increasing velocity of air, the wind power also increases. For an increase in velocity of air from 4 m/s to 8 m/s the wind power increases from 0 W to 11 MW. The relation can be observed as power increases at an increasing rate since the power output is proportional to the cube of velocity. Most of the power generated from the wind turbine is consumed in electrolysis at around 70%. The remainder is used to power the air conditioning cycle, refrigeration cycle, and heat pump cycle.

**Figure 10:**
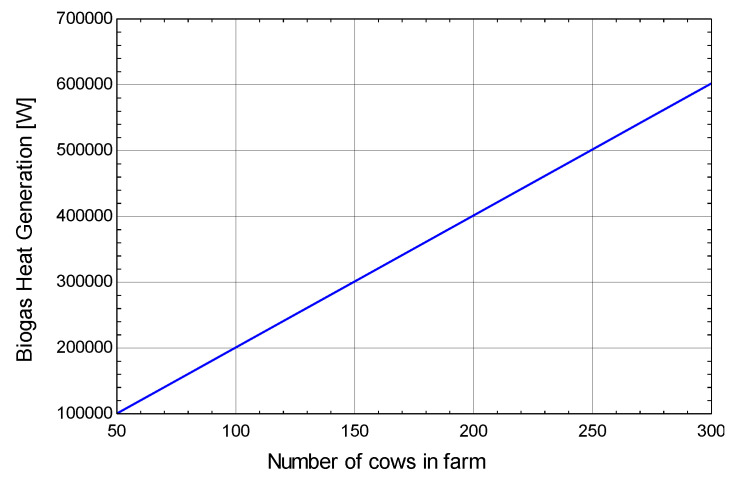
Effect of number of cows on biogas heat generation.

Figure 10 shows the relationship between the number of cows on a farm and biogas heat generation using the blue line. On increasing the number of cows on the farm, biogas heat generation also increases. For an increase in the number of cows from 50 to 300 the biogas heat generation increase from 0.1 MW to 0.7 MW. Biogas is used to provide heat input to boilers at the single-stage desalination and water treatment plant.

**Figure 11:**
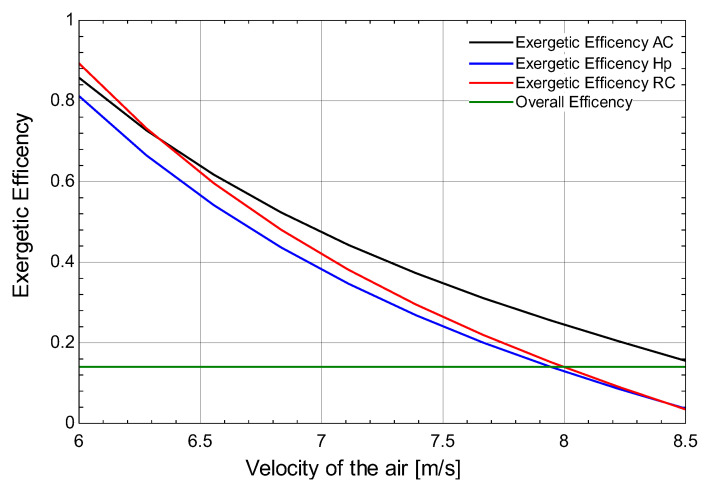
Effect of the velocity of the air on exergetic efficiency of the air-conditioning cycle, heat pump, refrigeration cycle, and overall exergetic efficiency.

Figure 11 shows the relationship between the velocity of the air and the exergetic efficiency of the air-conditioning cycle, heat pump, refrigeration cycle, and overall exergetic efficiency. It is seen that on increasing the velocity of the air that the exergetic efficiency of the air-conditioning cycle, heat pump, and refrigeration cycle decreases, whereas the overall exergy remains the same. For an increase in the velocity of air from 6 m/s to 8 m/s, the exergetic efficiency varies between 0 to 1.

**Figure 12:**
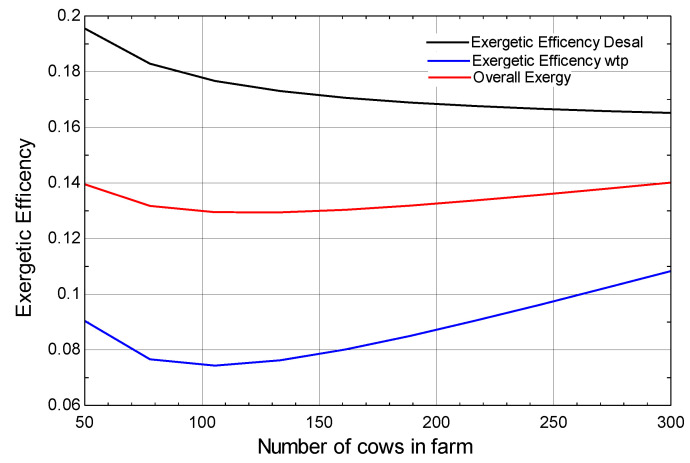
Effect of number of cows on exergetic efficiency of the desalination plant, water-treatment plant, and overall exergetic efficiency.

Figure 12 shows the relationship between the number of cows on the farm and the exergetic efficiency of the desalination plant, water treatment plant, and overall exergetic efficiency. It can be seen that on increasing the number of cows on the farm from 50 to 120 the overall exergy decreases but later it increases from 120 to 300 and there are two sources which are being powered; one is the desalination plant and the other is the water treatment plant. Overall exergy follows the same trend as for desalination and water treatment. In this case the velocity of the air is kept constant which results in none of the vapor compression cycle varying.

**Figure 13:**
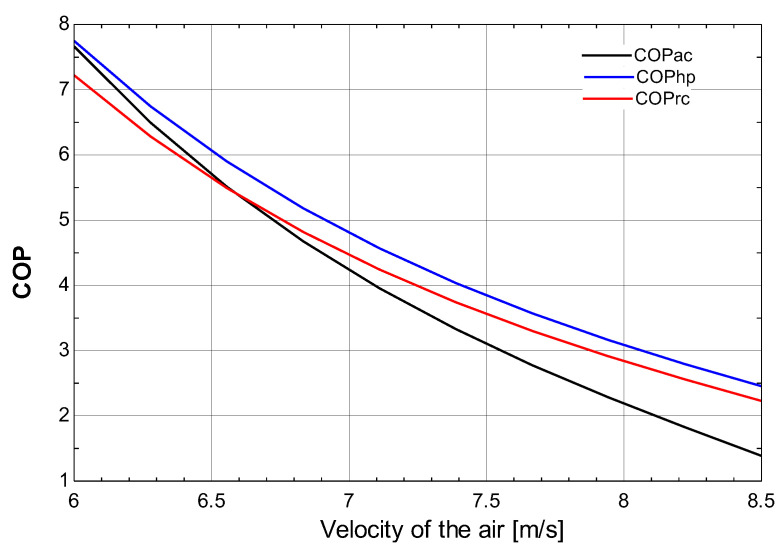
Effect of the velocity of the air on COP of heat pump, refrigeration cycle, and air-conditioning cycle.

Figure 13 shows the relationship between the velocity of air and the COP of the air-conditioning cycle, heat pump, and refrigeration cycle. It can be seen that on increasing the velocity of the air the COP of the air-conditioning cycle, heat pump, and refrigeration cycle decreases. For an increase in the velocity of air from 6 m/s to 8 m/s, the COP decreases from 8 to 1.

**Figure 14:**
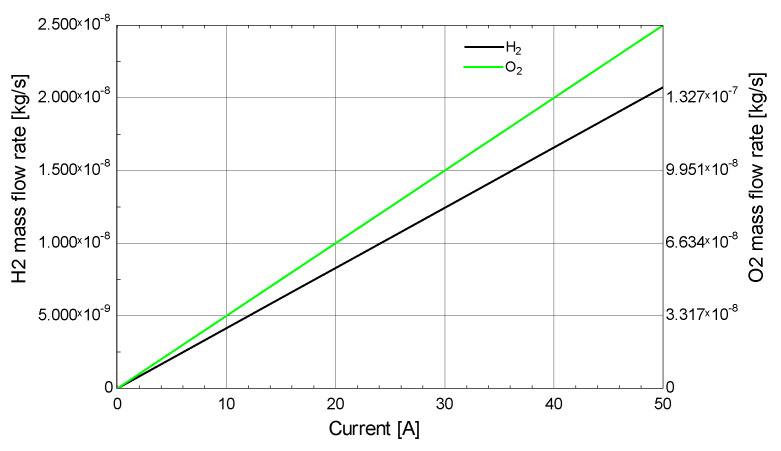
Effect of current on the mass flow rate of hydrogen.

Figure 14 shows the relationship between the current and mass flow rate of hydrogen. Most of the power supplied by the wind turbine is consumed for hydrogen production, hence the maximum current is produced; the voltage is kept constant at 2 V for the electrolysis process. It can be observed that with increasing the current the mass flow rate of hydrogen increases. For an increase in current from 0 A to 50 A the mass flow rate of hydrogen increases from 0 to 1.3 × 10^−7^ kg/s black line. The mass flow rate of oxygen also increases from 0 to 2.5 × 10^−8^ kg/s green line.

**Figure 15:**
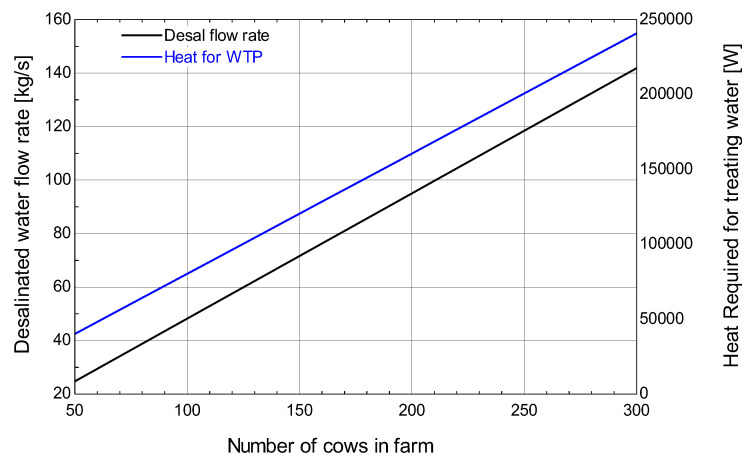
Effect of number of cows on a farm on desalinated water flow rate.

Figure 15 shows the relationship between the number of cows on the farm and the desalinated water flow rate. It can be seen that with increasing the number of cows on the farm the desalinated water flow rate also increases. For an increase in the number of cows on a farm from 50 to 300 the desalinated water flow rate increases from 20 kg/s to 160 kg/s.

**Figure 16:**
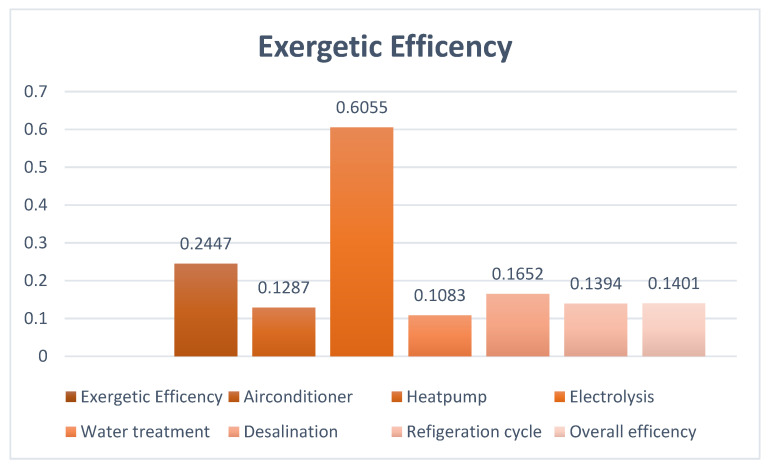
Exergy efficiency of different components.

Figure 16 shows the exergy efficiencies of different processes. It can be seen that air-conditioning cycles have the highest exergy efficiency and electrolysis has the lowest exergy efficiency. The exergy efficiencies of air-conditioning, desalination plant, electrolysis process, heat pump, refrigeration cycle, water treatment plant, and overall exergy efficiency are 0.2447, 0.1652, 0.6055, 0.1287, 0.1394, 0.1083, and 0.1401 respectively.

The root locus method was used to study the stability of the system. If for a bounded input, there is bounded output then the system is said to be stable. If all the poles lie on the right-hand side of the poles zero graphs, then the system is stable. If even a single pole lies on the left-hand side of the graph the system is said to be unstable. Root locus varies the gain of the system and plots the poles zero graphs; it allows the user to analyze and identify the parameters of the control system within which the system should be operated. A transfer function could be either a first-order system, second-order system, or at maximum a third order system for the multigeneration cycle. Hence for heat pumps, air conditioners, refrigeration cycle, and electrolysis, a first-order system is used, while for water treatment, desalination, and for power generation a cycle zero-order system is used.

A PID controller is used to stabilize the system response and make the system respond quickly. The PID autotune function is available in MATLAB which provides the best proportional, integral, derivative, and filter coefficient values for the system. In the case of the air conditioner, these values are P = 1.338, I = 0.08912, D = −1.457, and N = 0.126. Figure 9 shows that earlier the tuned response system was highly unstable and the thermostat was not maintained until 100 s, however, afterwards the tuning system can achieve a steady state in 60 s with negligible oscillations.

**Figure 17:**
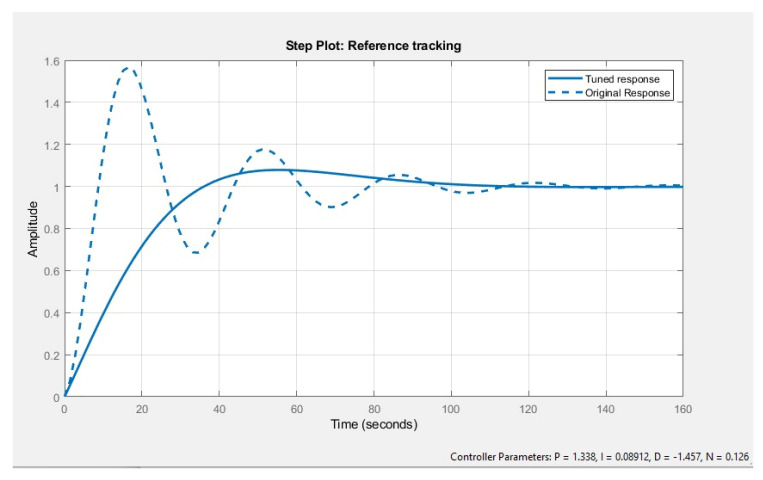
Time response of vapor compression cycle before tuning and after tuning.

**Figure 18:**
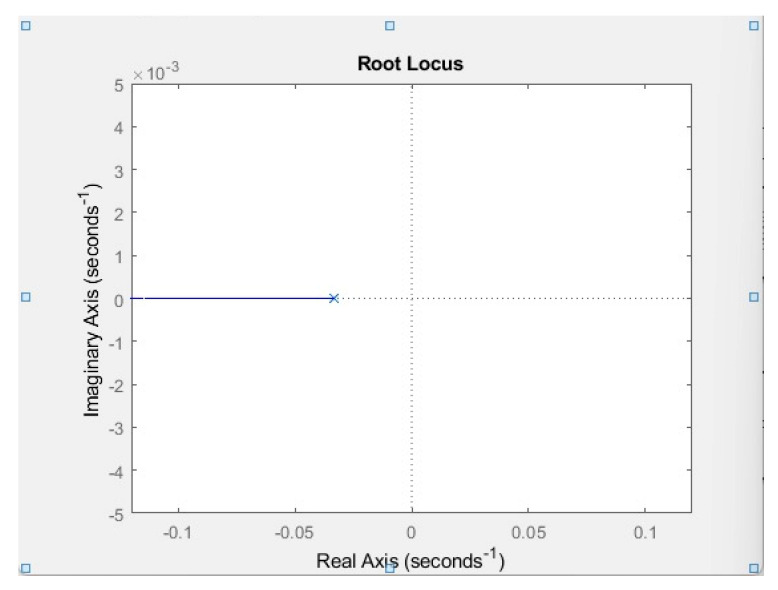
Root locus plot of vapor compression cycle.

In the air-conditioner, refrigeration cycle, and heat pump the first-order system was considered. Figure 17 shows that when the root locus is plotted, it results in a horizontal line on the left side of the zero poles graph. Because all the poles are lying on the right-hand side of the graph then any range of mass flow rates could be applied across these sub-cycles hence the system is stable at any available point. In a study conducted by [28], the response time of the compressor was experimentally and mathematically calculated, and the time constant for the air compressor was observed to be 30 s. Hence 30 s was used as the time constant for the refrigeration cycle transfer function. Figure 18 shows the root locus plot of the vapor compression cycle.

**Figure 19:**
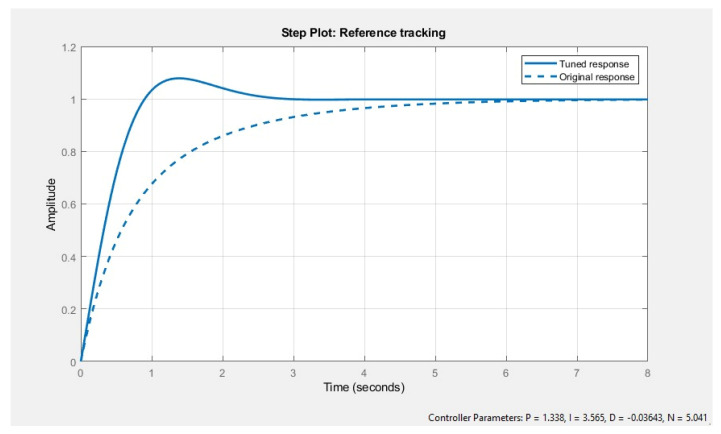
Time response of electrolysis system before tuning and after tuning.

For the electrolysis transfer function, the first order system was applied. Electrolysis is performed by supplying DC to a brine solution which results in the production of hydrogen and oxygen gas. The PID was also integrated with electrolysis by Simulink. After tuning, the electrolysis achieved a faster system response and tuned values were obtained as in Figure 19.

Battery time response has been studied in multiple literature examples. This multigeneration cycle time constant of 0.75 s is in reference to the study by [29] which is used in the Simulink first-order transfer function. Figure 11 shows PID tuned response; obtained values for the tuned response were P = 1.338, I = 3.565, D = −0.03643, and N = 5.041. Before, the tuned response system reaches a steady state at 4 s, afterwards the tuned response system can achieve a steady state at 2 s for the electrolysis system.

**Figure 20:**
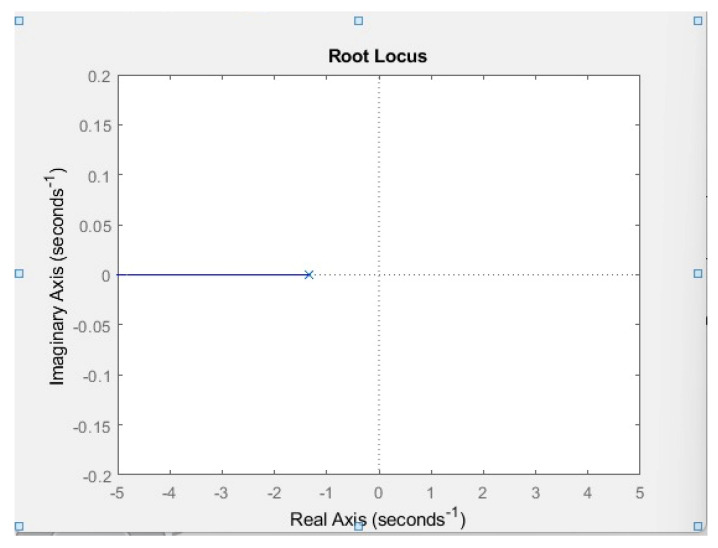
Root locus plot of electrolysis system.

Figure 20 shows that when the root locus plot is plotted a horizontal line on the left-hand side of the real axis is generated. Since none of the poles lies on the right side of the pole zero plot, the system is considered stable.

There were several key finding and results obtained from this study. Overall exergetic efficiency for operating this multigeneration cycle was equal to 0.1401. For having wind airspeed of around 7–8 m/s and using biogas produced from the manure of 200–300 cows in a farm, 140 kg/s desalinated water flow rate was obtained, while 5 × 10^−7^ kg/s of hydrogen gas, and 5 × 10^−6^ kg/s of oxygen gas were obtained from the electrolysis. The Simulink steady state thermodynamic model was built successfully. In addition, tuned PID parameters were obtained for the individual cycles, and the root locus graph was plotted to determine the stability of the thermodynamic cycles. 

## 4. Discussion

The method used to analyze the multigeneration cycle was performed using the methodology of previous literature. However, the parametric and requirements for this system were different from other studies. To validate the study performed, the system was modeled using the available parameters from the studies. After a comparison of the results, the percentage differences for the gas production, power input, power output, and exergy efficiency from the available literature were obtained. Table 2 shows deviation of the results for the calculations from different sources. Since most of the values are close to each other, the model used tended to generate accurate results like the other similar studies conducted. For the vapor compression cycle, the difference in exergy efficiency is 0.0% from Cengel’s [27] vapor compression cycle model. Similarly, for the other cycles like wind power, biogas production, and electrolysis the difference from the literature is negligible. For desalination the thermodynamic model prepared is novel, for this reason energy and exergetic analysis comparison isn’t available N/A; this analysis can only be determined by changing the operational standard. A limited library is available for thermodynamic modeling in Simulink. In this study the multigeneration system for sustainable cities was thermodynamically analyzed using both EES and Simulink software. Additionally, the best transfer function obtained for the individual components from the literature was used to obtain steady state response through tuning of the PID controller. Finally, a root locus plot was provided to identify the stability of the processes of the multigeneration system.

## 5. Conclusions

For this proposed multigeneration cycle, wind and biogas were used as power generation. For the energetic and exergetic analyses, the cycle was modeled on EES software. The combined exergetic efficiency was equal to 14%; the major contributor to the loss of overall exergy was due to biogas since most of the energy obtained from the biogas is lost. Other results included the COP of the refrigeration cycle and heat pump. The amount of hydrogen and oxygen produced was also calculated for different current cycles. Fresh water was produced using the desalination process and hot water was produced using the heat from the heat exchangers. The cycle was also modeled in Simulink using Python integrated into MATLAB to use the Cool Prop library. After identifying the expected order of the system for each component of the cycle, components were tuned for the PID controller while root locus plots were obtained. Currently, there is a limited library available for thermodynamic modeling in Simulink and for other programming software. The one which is available does not provide accurate readings for some of the refrigerants/working fluids. For future work, the transfer functions could be obtained from the actual compressor, pump, boiler, and heat exchanger to improve accuracy. A better controller could be introduced to improve tuning. In addition, the Simulink model could be improved to model a more complex cycle.

## Figures and Tables

**Table 1:** Parametric table of multigeneration system.

Parameter Value of Multigeneration System	
Wind turbine	
Velocity of the air (V)	8 m/s [24]
Density of the air (p)	1.20 kg/m^3^ [24]
Electrical losses	1%
Electricity transmission losses	1.25%
Mechanical losses	3%
Wake losses	5%
Biogas production	
Waste disposal of cow manure per day	20 kg [25]
Volatile solid cow manure per day	1.42 kg [25]
Yield factor	5.5 [25]
Electrolysis	
Voltage for electrolysis (V)	2 V
Energy of hydrogen gas at r.t.p	116648 kJ/k [26]
Energy of oxygen gas at r.t.p	24.68 kJ /kg [26]
Faraday’s constant	96485
Vapor compression cycle	
Ambient temperature (To)	25 °C
Compressor isentropic efficiency (n)	0.85
Refrigeration cycle low pressure side (Prc)	140 kPa [27]
Heat pump low pressure side (Php)	140 kPa [27]
Air conditioner low pressure side (Pac)	400 kPa
Refrigerant used for vapor compression	R410a
Mass flow rate of refrigeration cycle (mrc)	14 kg/s
Mass flow rate of the heat pump (mhp)	15 kg/s
Mass flow rate of the air conditioner (mac)	19 kg/s

**Table 2:** Validation table for the results obtained.

	Mass H_2_/Power Input/Power Output			Exergy Efficiency			Source
	Present	Published	Difference	Present	Published	Difference	
Vapor Compression Cycle	1.81	1.82	0.5%	56%	56%	0.0%	[27]
Wind Power	8100	7792	4.0%	0.8607	0.845	1.8%	[30]
Biogas Production	9430	9391	0.4%	N/A	N/A	N/A	[25]
Electrolysis	0.06561	0.06562	0.015%	0.6054	0.6055	0.017%	[31]
Desalination	N/A	N/A	N/A	N/A	N/A	N/A	[26]

## Data Availability

The data presented in this study are available on request from the corresponding author.
